# Up in smoke? Limited evidence of a smoking harm paradox in 17-year cohort study

**DOI:** 10.1186/s12889-023-16952-6

**Published:** 2023-10-17

**Authors:** Samantha Marie Harris, Magnus Jørgensen, Emily Lowthian, Sara Madeleine Kristensen

**Affiliations:** 1 Department of Health Promotion and Development, University of Bergen, Alrek helseklynge, Årstadveien 17, Bergen, 5009 Norway; 2 Department of Education and Childhood Studies, Swansea University, Singleton Campus, Swansea, Wales SA2 8PP UK

**Keywords:** Disparities, Socioeconomic status, Smoking caused Disease

## Abstract

**Background:**

Socioeconomic differences in the impact of alcohol consumption on health have been consistently reported in the so-called “alcohol harm paradox” (i.e., individuals from higher socioeconomic backgrounds (SES) drink more alcohol than individuals from lower SES, but the latter accrue more alcohol-related harm). Despite the severe health risks of smoking however, there is a scarcity of studies examining a possible “smoking harm paradox” (SHP). We aim to fill this gap.

**Methods:**

We conducted a prospective cohort study with adolescents from the Norwegian Longitudinal Health Behaviour Study (NLHB). Our study used data from ages 13 to 30 years. To analyse our data, we used the random-intercept cross-lagged panel model (RI-CLPM) with smoking and self-reported health as mutual lagged predictors and outcomes as well as parental income and education as grouping variables. Parental income and education were used as proxies for adolescent socioeconomic status (SES). Smoking was examined through frequency of smoking (every day, every week, less than once a week, not at all). General health compared to others was measured by self-report.

**Results:**

Overall, we found inconclusive evidence of the smoking harm paradox, as not all effects from smoking to self-reported health were moderated by SES. Nevertheless, the findings do suggest that smoking predicted worse subjective health over time among individuals in the lower parental education group compared with those in the higher parental education group. This pattern was not found for parental income.

**Conclusions:**

While our results suggest limited evidence for a smoking harm paradox (SHP), they also suggest that the impact of adolescent smoking on later subjective health is significant for individuals with low parental education but not individuals with high parental education. This effect was not found for parental income, highlighting the potential influence of parental education over income as a determinant of subjective health outcomes in relation to smoking.

**Supplementary Information:**

The online version contains supplementary material available at 10.1186/s12889-023-16952-6.

## Background

The harmful effect of social disadvantage on health, in other words social inequities in health, have been reported consistently in previous literature and are long-lasting and significant [1, 2]. Despite being hailed as an equal society, Norway also shows evidence of social inequities in health. For example, men and women with the highest level of attained education live approximately 5–6 years longer than those with the lowest educational attainment [3], and differences in mortality between educational groups in Norway are among some of the largest in Europe [3, 4].

It has been suggested that social inequities in health can be attributed in part to differences in lifestyle behaviours, such as smoking. According to the Norwegian Directorate of Health [5] and Statistics Norway [6], 9% of people in Norway between the ages of 16–74 years smoked daily in 2020, and 6,300 people die of tobacco related diseases each year making smoking an important public health concern. While some studies suggest that daily smoking among youth is decreasing [7], the health effects of smoking earlier in life are likely to still be unfolding today. Smoking has been linked to a wide range of health problems including but not limited to cancer, respiratory and cardiovascular diseases, inflammation, impaired immune function [8], and mental health problems [9]. The Norwegian Directorate of Health [5] reports that individuals with no to low educational attainment have a higher prevalence of smoking than those with higher educational attainment. Similarly, a study conducted among Norwegian adolescents aged 16 to 20 found that those who had no plans to undertake further education versus those who did, had up to 3.8 higher odds of smoking [10]. Furthermore, a systematic review exploring smoking and social inequities concluded that people from disadvantaged backgrounds were both more likely to smoke and experience severe harms from smoking, for example becoming trapped in poverty [11].

Hence, it appears that people from lower socioeconomic backgrounds may incur more severe health harms from certain substances. Alcohol use, for example, has been associated with greater health concerns in lower socioeconomic groups [12]. This has been described in the “alcohol harm paradox” (AHP), which posits that individuals from lower socioeconomic backgrounds experience disproportionately greater alcohol-related health harms than individuals from higher socioeconomic background at the same, or lower, levels of alcohol consumption [13]. The AHP has been reported consistently in previous literature [13–15]. This has been shown in studies for both adults and adolescents using cross-sectional and longitudinal designs [16, 17]. Research regarding a “smoking harm paradox” (SHP), on the other hand, is scarce.

A life course perspective suggests that it is necessary to measure the early precursors of diseases to fully grasp their development over time [18]. Onset of smoking commonly occurs during adolescence [19], however, some smoking-related diseases endure a long latency period before manifesting clinically [20, 21]. This suggests that clinical measures of smoking-related harm might not be appropriate at earlier stages of disease development (e.g., adolescence), instead, self-reported health might be more indicative of how adolescent smoking affects health at the subclinical level. The discovery of a SHP as based on self-reported health would corroborate this line of thinking and highlight adolescence as a key developmental period for early interventions aiming to not only reduce smoking but also long-term smoking-related health disparities [19].

## Aims

Thus, the purpose of the current study is to investigate a possible SHP in a longitudinal dataset of Norwegian youths (NLHB) that were followed up over 17 years. Though earlier studies using the NLHB have looked at smoking in relation to various health outcomes (e.g., depression) and using different predictors (e.g., sibling smoking, parental smoking etc.), none have specifically considered a smoking-related harm paradox and if this association can be detected at a pre-clinical level as the harmful effects of smoking gradually unfold from adolescence to adulthood [21–24]. Our research question is whether the relationship between smoking frequency and self-reported health is moderated by parents’ educational attainment and income (as proxies for childhood SES). If the harm paradox holds for smoking, we hypothesise that individuals who smoke from higher socioeconomic backgrounds would report better self-reported health than those with lower socioeconomic backgrounds.

## Methods

### Study design

We conducted a prospective cohort study using data from the ‘Norwegian Longitudinal Health Behaviour Study’ (NLHB), in which a cluster sample from a cohort of adolescents was followed from age 13 (1990) to 30 (2007). See Table 1 for the sample size of the study between 1990 and 2007. The NLHB study was reviewed by the Data Inspectorate of Norway and received a recommendation from the Regional Committee of Medical Research Ethics (REK). Informed written consent has been obtained from participants at every consecutive time point. More detailed information on data collection is available in previous publications [22, 25]. See Appendix A for a frequency table of variables of interest at each time point. Other studies using the NLHB dataset have also reported on attrition - showing that individuals with higher levels of parental income and education are more likely to participate at later follow-ups [26]. For the present study, we also conducted an attrition analysis for participation in 2007 (age 30) but did not find any associations with degree of smoking and self-reported health at baseline in 1990 (age 13) (See Appendices). For the NLHB total sample, 43% (n = 536) participated in 2007 (age 30) from the original n = 1242 which participated at least once during the study period (See Table 1 for frequencies).

**Table 1:** Sample size at each timepoint between 1990–2007. N refers to the number of respondents. Note that newly enrolled students at the invited schools were invited to participate during the first waves of the study which increased the total sample size to at least 1242 unique individuals who have participated once

Year	1990	1991	1992	1993	1995	1996	1998	2000	2007
Age	13	14	15	16	18	19	21	23	30
N	924	958	963	789	779	643	634	627	536

### Variables

#### Outcome

##### Self-reported health

In line with previous literature, which has used single item measures to suggest that smokers typically report worse self-perceived health than non-smokers [27–30] and because of the wide range of health problems associated with smoking, we chose to focus on participants’ self-reported health. We measure self-reported health through the item ‘How would you rate your health compared to others your age?’. Response options were: ‘very good’, ‘good’, ‘neither’, ‘bad’ and ‘very bad’.

#### Predictor

##### Smoking frequency

Participants’ smoking frequency was measured through the item ‘How often do you smoke?’ with response options: ‘every day’, ‘every week’, ‘less than once a week’ or ‘not at all’. Similar wording has been used to examine smoking frequency in previous studies [27, 31].

#### Groups

##### Socioeconomic status

Given that our baseline measurement was taken when participants were aged 13 years, we measured adolescent socioeconomic status (SES) for 1995 through parents’ self-reported salary and educational attainment in 1996. The income variable has the following response categories: “Less than NOK 100.000”, “NOK 100–199.000”, “NOK 200–299.000”, “NOK 300–399.000”, “NOK 400–499.000” and “NOK 500.000 or more” with the annual average wage being 213 000 NOK in 1995 (1 NOK ≈ 0.12 EUR using the 2007 exchange rate). The education variable had the following response categories: “0 years of education after elementary school”, “1–2 years of education after elementary school”, “3 years of education after elementary school”, “Less than 4 years at university/college”, “More than 4 years at university/college” and “Other”. The last category and missing values were replaced with adolescents’ report of parental socioeconomic status following the logic described in Jørgensen and colleagues [26]. Both variables were dichotomised for use in the analyses, resulting in approximately equal distributions for parental education (low = 578, high = 390) and parental income (low = 323, high = 292). The NLHB dataset also contains data on adolescents’ report of parental education and occupation [25], however, using parental SES as a proxy for adolescent SES is a common procedure [32–34]. We also chose not to include parental occupation, as salaries and educational attainment are comparable across professions, while occupation can vary greatly in terms of responsibilities and pay.

### Control variables

We included gender as a control variable, given the differential educational gradient previously observed between men and women [35].

### Statistical analyses

Analyses were performed in Mplus 8 for Windows [36] using maximum likelihood estimation with robust standard errors to account for skewness in the data. Code is available on OSF (DOI 10.17605/OSF.IO/ZCBMS). We conducted a random intercept cross lagged panel model (RI-CLPM) of the relationship between self-reported health and smoking frequency (i.e., every day, every week, less than once a week, not at all) with parental education and income as a moderator. The RI-CLPM was specified following Hamaker [37]. We used standard cut-offs for evaluation of fit for structural equation models: CFI: ≥ 0.90, RMSEA: ≤ 0.08 and SRMR: ≤ 0.08 [38, 39]. Our model evaluation did not rely on the p-value for the chi-square test as studies have shown this is too sensitive to sample size, and thus, not an appropriate criterion for evaluation [40]. We used Full Information Maximum Likelihood (FIML) to manage bias associated with missing data.

**Fig. 1:**
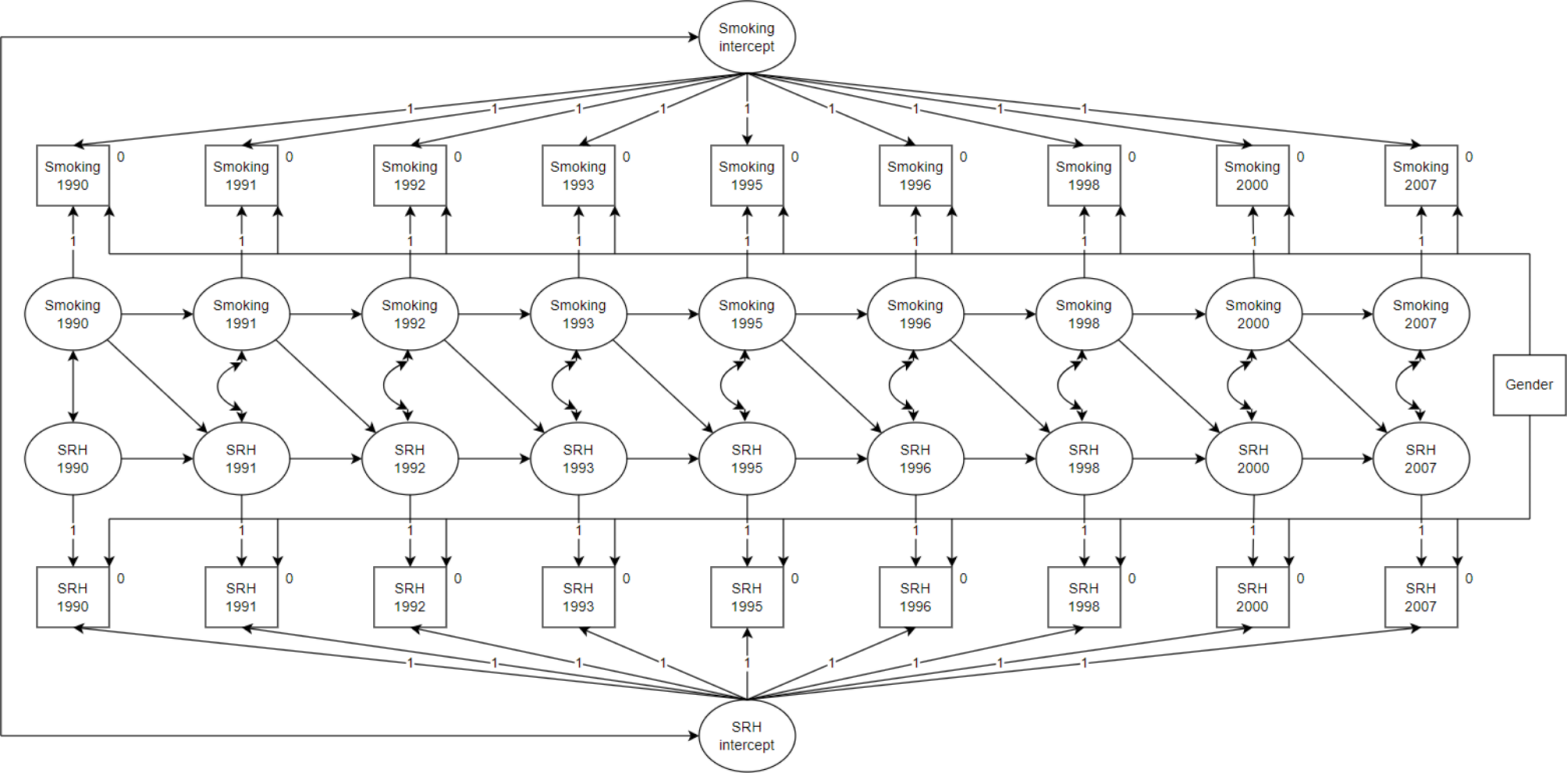
RI-CLPM specification of smoking and self-reported health. *Note*. SRH = self-reported health

See Fig. 1 for the measurement model. Initially, we created two random intercepts, one for each construct, with factor loadings to all time points constrained to unity. Next, we specified 18 latent within-person variables: one for each measurement occasion in both constructs across nine time points. These latent variables had factor loadings constrained to unity. We constrained the variance in all observed variables to zero to ensure all variance is captured by the intercepts and within-person variables. Next, we added cross-lagged effects from smoking to self-reported health. Notably, we did not include cross-lagged effects from self-reported health to smoking as these effects were not directly relevant to our hypothesis. In addition, we wanted to make our model as parsimonious as possible with as few parameters to estimate as needed.

To investigate whether the model had time-invariant effects from smoking to self-reported health, we constrained similar time intervals of the cross-lagged effects to be equal throughout the study (e.g., 1990 to 1991/1991 to 1992 and 1993 to 1995/1996 to 1998). We compared the model fit of a freely estimated model to the model with cross-lagged constraints using a chi-square difference test. This was examined in all socioeconomic status groups: low parental education, high parental education, low parental income, and high parental income. If the model fit improved or did not significantly deteriorate in the nested model compared to the freely estimated model, the cross-lagged constraints were deemed tenable and kept in place for the multi-group analyses. Lastly, gender was added as a control variable with regression coefficients to the observed variables on each time point.

The socioeconomic moderation analyses were performed using multi-group analyses across parental education and income with 1000 bootstraps. Cross-lagged parameters were compared across groups using the model constraint function in Mplus.

Our analyses were preregistered on the Open Science Framework. The original pre-registration was published on the 7 February 2023 (10.17605/OSF.IO/U9XVR), and the edited version following changes in analysis due to failed model fit procedures was published on 6 March 2023 (10.17605/OSF.IO/9Z27A).

## Results

### Socioeconomic moderation RI-CLPM of smoking and self-reported health

The time-invariant cross-lagged constraints did not significantly deteriorate in the low (ΔX^2^ = 3.39, Δ*df* = 5, *p* = .640) and high (ΔX^2^ = 1.87, Δ*df* = 5, *p* = .867) parental education groups. Similarly, the cross-lagged constraints did not significantly worsen model fit in the low (ΔX^2^ = 6.21, Δ*df* = 5, *p* = .286) and high (ΔX^2^ = 1.88, Δ*df* = 5, *p* = .865) parental income groups. Thus, we kept the time-invariant cross-lagged constraints in the multi-group analyses. For space constraints, we only present the standardised cross-lagged effects of smoking on self-reported health across socioeconomic groups. Please see Appendix B for a comprehensive overview of the results.

### Parental education

**Fig. 2:**
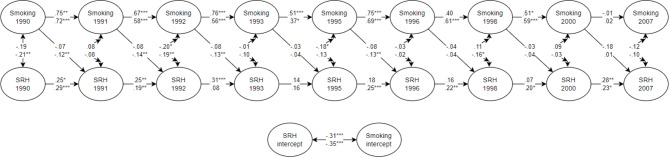
Simplified presentation of the parental education moderation of the RI-CLPM with Smoking and self-reported health. *Note*. Standardised estimates are presented in the figure. The high parental education group is on the top line and the low parental education group is on the lower line. *** p < .001, ** p < .01, * p < .05. SRH = self-reported health

The parental education multi-group RI-CLPM with smoking and self-reported health achieved acceptable model fit: X^2^ = 427.065, *df* = 252, RMSEA (95% CI) = 0.040 (0.033 – 0.047), CFI = 0.962, SRMR = 0.062. The results are presented in Fig. 2 which demonstrate the association from smoking to self-reported health, associations between smoking and self-reported health at the same time-point, an estimate for the association between smoking at one time-point to the next, and the estimate of the association of the random intercepts between smoking and self-reported health (*β* = − 0.31 and − 0.35). Although there are some cross-lagged differences from smoking to self-reported health across parental education groups, these differences were not significant. The low parental education group had negative and significant cross-lagged effects from smoking to self-reported health from 1990 to 1991 (*β* = − 0.12, *p* < .01), 1991 to 1992 (*β* = − 0.14, *p* < .01), 1992 to 1993 (*β* = − 0.13, *p* < .01), and 1995 to 1996 (*β* = − 0.13, *p* < .01). There were no significant cross-lagged effects from smoking to self-reported health in the high parental education group.

### Parental income

**Fig. 3:**
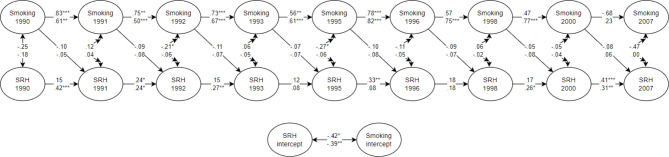
Simplified presentation of the parental income moderation of the RI-CLPM with Smoking and self-reported health. *Note*. Standardised estimates are presented in the figure. The high parental income group is on the top line and the low parental income group is on the lower line. *** p < .001, ** p < .01, * p < .05. SRH = self-reported health

The parental income multi-group analysis of the RI-CLPM with smoking and self-reported health produced acceptable model fit: X^2^ = 3.85.816, *df* = 252, RMSEA (95% CI) = 0.044 (0.035 – 0.052), CFI = 0.957, SRMR = 0.064. The results are presented in Fig. 3, which demonstrate the association from smoking to self-reported health, associations between smoking and self-reported health at the same time-point, an estimate for the association between smoking at one time-point to the next. There were no discernible differences between the parental income groups on the cross-lagged effects from smoking to self-reported health. There were no significant cross-lagged effects from smoking to self-reported health in either of the parental income groups.

## Discussion

We find inconclusive evidence of a smoking harm paradox in this study. Our findings suggest that young people aged 13–19 years whose parents had lower educational attainment experienced negative effects of smoking on later self-reported health in the period from 1990 to 1996. Based on a simulation study by Orth [41], which categorised the size of effects in RI-CLPM analyses, we can further conclude that the statistically significant effects were large. Contrastingly, the effects of smoking on subsequent self-reported health in the high parental education group were small to moderate [41] and not significant. Smoking did not significantly affect later self-reported health among people in the high or low parental income groups. Furthermore, the difference between the groups in a moderation analysis was not statistically significant.

Our mixed comparative findings imply *some* similarities to those implied by the alcohol-harm-paradox. While significant associations were identified, they were only captured in relation to parental educational attainment, rather than parental income, suggesting the paradox may be sensitive to specific dimensions of socioeconomic status. Socioeconomic status is a complex social construct, and when measured differently often captures associations of differing size or directions. Educational qualifications offer knowledge assets, and at a parental level offer choice and constraints on how their children’s socioeconomic circumstances can be influenced, including education which is a strong determinant of employment and income opportunities [42–44]. Importantly, the predictive power of education has previously been highlighted by Winkleby and colleagues [45], who concluded that higher education may be the strongest SES predictor of good health. Whereas income relates to material assets [43], which *can* be used to gain access to health promoting environments, such as green-space, safe employment, and commodities such as food, exercise along with a higher relative social-standing [42]. In a study of adolescents, Melotti and colleagues [12] found that a higher socioeconomic position was associated with decreased previous and ever smoking, notably maternal education showed a greater effect size compared to disposable income. In the case of smoking, we find that parental educational attainment is a key construct of socioeconomic status which is likely to capture attitudes, knowledge, and general acceptability of smoking. Hence, further research must explore the smoking harm paradox with a representative sample utilising multiple constructs of socioeconomic status (i.e., education, income, neighbourhood) to examine the differing contributions of social, cultural, and economic advantage.

While our study did not examine underlying mechanisms, it is possible that some mechanisms that have been proposed for the AHP may also be relevant here. For example, populations being exposed to other health challenges that interact creating a ‘multiplicative’ risk to health [46]. It is well known, for instance, that smoking is positively associated with a variety of health behaviours during adolescence, including consumption of alcohol, soft drinks and fast food [47]. In a study of Icelandic adolescents, it was found that *some* health behaviours had a multiplicative risk, with diet, physical activity and substance use constituting separate constructs using principal component analysis [48]. Similarly, a more recent study of Italian adults found *some* health behaviours clustered together, although some also had a mixed picture of healthful and unhealthful behaviours [49]. This multiplicative risk may occur at multiple levels including individual, neighbourhood (access to healthful food, or areas to exercise), structural, or political (funding afforded to area, policies to support deprived groups etc.) [50]. Other mechanisms include lifestyle, suggesting that differences in health behaviours observed between socioeconomic groups may be the result of strong socioeconomically patterned environmental influences (e.g., high levels of social and physical stressors) with ramifications for general health consciousness [14]. In essence, it is possible that the paradox represents engagement in wider health behaviours which could extend smoking or alcohol. Further research must confirm this with a longitudinal, representative samples of adolescents.

The results of our study should also be considered in the international context. Norway has consistently been “one step ahead” of the recommendations and regulations of the EU and World Health Organisation Framework Convention on Tobacco Control (WHO FCTC) [51], with a ban on the advertising of tobacco products being in 1973, compared to 1978 in Finland [52, 53], 2002 in the UK, and 2009 in Ireland (despite being signed in 2002) [54]. In Norway, smoking rates have decreased at a faster rate compared to some other EU countries, particularly for men at low and high educational levels [55]; in 2019 only 10% of the population in Norway were considered ‘daily smokers’ compared to the EU average of 18% [56]. Moreover, given Norway has a Gini coefficient of 0.26 [57], the fifth lowest income inequality in the entire index, it is plausible that the paradox could be a larger problem in other, less equal countries, particularly those with limited tobacco control, although it must be noted that there is unclear evidence regarding the equity impact of tobacco control interventions [58]. However, while Norway has progressive tobacco control measures in a global context, we still observed some indication of a paradox in the adolescent years; this association could be due to adolescent-specific symptoms, policy changes, or our sample being underpowered at older ages. We recommend that future research investigate the smoking harm paradox, with specific focus on the effect of parental education, across multiple countries to examine its generalisability beyond the Norwegian context. We further recommend that future research include other measures of sub-clinical health that capture the early effects of smoking.

### Strengths and limitations

The study’s strengths include the panel design and follow-up time of 17 years, which allowed us to examine intra-individual changes over time. However, one possible reason for our inconclusive results is the limited variation in SES among our participants. Future studies should examine a smoking harm paradox in more socioeconomically heterogeneous samples across different countries as Norway is known to have a compressed wage structure - with reduced income-gains by additional years of education [56, 59]. Furthermore, we have based our analyses on self-reported measures of smoking. Self-report measures have been criticised for being unreliable and at risk of under-reporting [11]. However, under-reporting of alcohol consumption is said to be similar across socio-economic groups [46] and while a tendency to underreport smoking status has been observed among low international SES women and male blue collar workers, the validity of self-reported smoking status did not differ by SES [60]. Hence, while people may generally under-report smoking, there is little reason to believe that this should differ systematically between socioeconomic groups. It has also been suggested that adolescents from higher SES backgrounds are more likely to participate at later follow-ups [61], a bias that may have affected our findings in that our findings for lower SES individuals may be less valid. In fact, earlier studies using the NLHB data do confirm a bias towards higher SES individuals (i.e., higher parental income and education) being more likely to participate at later time points [26]. Finally, SES is a complex concept and the way in which we chose to measure it, through parental education and salary rather than occupation, may have had impacts on our findings. We present our justification for these choices, but future studies may want to consider different measures of SES as discussed in Cohen [62]. More studies are needed to conclude whether and under what circumstances a smoking harm paradox exists. Finally, we should also note that although the present study uses data dating back to 1990, we believe the longitudinal dimension of the data with multiple time points outweighs the cohort-specific limitations that might be present by adding a novel contribution to the research field.

## Conclusions

We found inconclusive evidence of SES affecting participants’ self-reported health over time. However, the findings suggest that the impact of adolescent smoking on later subjective health is significant for individuals in the low parental education group but not individuals in the high parental education group. This pattern was not found for parental income. These findings suggest that the smoking harm paradox might be tentatively comparable to the alcohol harm paradox. Our study lays the groundwork for future studies and indicates that education may play a different role in the impact of smoking on self-reported health compared to income. Nevertheless, this should be further examined in different contexts before clear conclusions can be drawn on the existence of the smoking harm paradox.

### Electronic supplementary material

Below is the link to the electronic supplementary material.

Supplementary Material 1

## Data Availability

Our analyses were preregistered on the Open Science Framework. The original pre-registration was published on the 7 February 2023 (10.17605/OSF.IO/U9XVR), and the edited version following changes in analysis due to failed model fit procedures was published on 6 March 2023 (10.17605/OSF.IO/9Z27A). Mplus syntax for the RI-CLPM analyses is available on OSF (DOI 10.17605/OSF.IO/ZCBMS). The data are available upon reasonable request as explicit consent for depository sharing hasn’t been obtained.

## Abbreviations

SES: Socioeconomic status
