# Supplementary material for: Up in smoke? Limited evidence of a smoking harm paradox in 17-year cohort study
Source: BMC Public Health. 2023 Oct 17;23:2022. doi: 10.1186/s12889-023-16952-6 (PMC10580607; doi:10.1186/s12889-023-16952-6)
Supplement: Supplementary file 1 — Supplementary Material 1 [file 12889_2023_16952_MOESM1_ESM.docx]

**Appendix A1.** *Frequency table of variables of interest.*

|  |  | 1990 | 1991 | 1992 | 1993 | 1995 | 1996 | 1998 | 2000 | 2007 |
| --- | --- | --- | --- | --- | --- | --- | --- | --- | --- | --- |
|  |  | N (%) | N (%) | N (%) | N (%) | N (%) | N (%) | N (%) | N (%) | N (%) |
| How often do you smoke? | | | | | | | | | | |
|  | Daily | 29 (2.3) | 82 (6.5) | 160 (12.7) | 154 (12.3) | 241 (19.2) | 217 (17.3) | 209 (16.6) | 216 (17.2) | 111 (8.8) |
|  | Weekly | 77 (6.1) | 150 (11.9) | 165 (13.1) | 88 (7.0) | 131 (10.4) | 109 (8.7) | 96 (7.6) | 96 (7.7) | 65 (5.2) |
|  | Never | 781 (62.1) | 717 (57.0) | 590 (46.9) | 464 (36.9) | 404 (32.1) | 314 (25.0) | 280 (22.3) | 313 (24.9) | 358 (28.5) |
|  | Missing | 370 (29.4) | 308 (24.5) | 342 (27.2) | 551 (43.8) | 481 (38.3) | 617 (49.1) | 672 (53.5) | 632 (50.3) | 723 (57.5) |
| Self-reported health | | | | | | | | | | |
|  | Very good | 98 (7.8) | 146 (11.6) | 198 (15.8) | 126 (10.0) | 119 (9.5) | 82 (6.5) | 60 (4.8) | 80 (6.4) | 76 (6.0) |
|  | Good | 301 (23.9) | 391 (31.1) | 482 (38.3) | 348 (27.7) | 397 (31.6) | 333 (26.5) | 312 (24.8) | 327 (26.0) | 304 (24.2) |
|  | Neither | 229 (18.2) | 273 (21.7) | 249 (19.8) | 210 (16.7) | 235 (18.7) | 212 (16.9) | 201 (16.0) | 197 (15.7) | 128 (10.2) |
|  | Bad | 13 (1.0) | 25 (2.0) | 20 (1.6) | 19 (1.5) | 21 (1.7) | 15 (1.2) | 14 (1.1) | 16 (1.3) | 23 (1.8) |
|  | Very bad | 6 (0.5) | 2 (0.2) | 2 (0.2) | 2 (0.2) | 4 (0.3) | 1 (0.1) | 0 (0.0) | 4 (0.3) | 2 (0.2) |
|  | Missing | 610 (48.5) | 420 (33.4) | 306 (24.3) | 552 (43.9) | 481 (38.3) | 614 (48.8) | 670 (53.3) | 633 (50.4) | 724 (57.6) |
| Parental education | | | | | | | | | | |
|  | Lower |  |  | 578 (46.0) |  |  |  |  |  |  |
|  | Higher |  |  | 390 (31.0) |  |  |  |  |  |  |
|  | Missing |  |  | 289 (23.0) |  |  |  |  |  |  |
| Parental income | | | | | | | | | | |
|  | Lower |  |  | 292 (23.2) |  |  |  |  |  |  |
|  | Higher |  |  | 323 (25.7) |  |  |  |  |  |  |
|  | Missing |  |  | 642 (51.1) |  |  |  |  |  |  |

**Appendix A2.** Independent sample t-tests for self-reported health and smoking at age 13 for participation versus non-participation at age 30 (2007)

| Participation | No |  | Yes | |  |  | |  | |  |  | |  | |  |
| --- | --- | --- | --- | --- | --- | --- | --- | --- | --- | --- | --- | --- | --- | --- | --- |
|  | *n* | Mean  (SD) | *n* | Mean  (SD) | Mean difference | | F-value | | Two-tailed p-value | | | t- value |  | df | |
| Self-reported health | 316 | 3.74  (.80) | 331 | 3.72  (.75) | .03 | | .93 | | .65 | |  | .46 | | 645 | |
| Smoking | 433 | 1.23  (.69) | 454 | 1.20  (.56)) | .05 | | 6.30 | | .23 | |  | 1.20 | | 885 | |

**Appendix A3.** Missingness for self-reported health and smoking at every time point

| **Time point** | **Self-reported health** | **Smoking** |
| --- | --- | --- |
| *Year(age)* | n(%) | n(%) |
| 1990 (13) | 610(49) | 370(29.4) |
| 1991 (14) | 420(33) | 308(25) |
| 1992 (15) | 306(24) | 342(27.2) |
| 1993 (16) | 552(44) | 551(44) |
| 1995 (18) | 481(38) | 481(38.3) |
| 1996 (19) | 614(49) | 617(49.1) |
| 1998 (21) | 670(53) | 672(54) |
| 2000 (23) | 633(50) | 632(50) |
| 2007 (30) | 724(58) | 723(58) |

**Appendix B.** *Results of the random intercept cross-lagged panel models of smoking and self-reported health moderated by parental education and income.*

**Parental education**

The smoking and self-reported health intercepts were negatively and moderately correlated in the low (*r* = -.35, *p* < .001) and high (*r* = -.31, *p* < .001) parental education groups. Regarding within-person associations between smoking and self-reported health, the low parental education group showed significant, negative, and small correlations in 1990 (*r* = -.21, *p* < .01), 1992 (*r* = -.19, *p* < .01), and 1998 (*r* = -.16, *p* < .001). The high parental education group had only one significant, negative, and small within-person association between smoking and self-reported health in 1992 (*r* = -.20, *p* < .05).

In the low parental education group, there were positive and significant carry-over stability effects in self-reported health from 1990 to 1991 (*β* = .29, *p* < .001), 1991 to 1992 (*β* = .19, *p* < .01), 1995 to 1996 (*β* = .25, *p* < .001), 1996 to 1998 (*β* = .22, *p* < .01), 1998 to 2000 (*β* = .20, *p* < .05), and 2000 to 2007 (*β* = .23, *p* < .05). In the same group, there were also positive and significant carry-over stability effects in smoking from 1990 to 1991 (*β* = .72, *p* < .001), 1991 to 1992 (*β* = .58, *p* < .001), 1992 to 1993 (*β* = .56, *p* < .001), 1993 to 1995 (*β* = .37, *p* < .05), 1995 to 1996 (*β* = .69, *p* < .001), 1996 to 1998 (*β* = .61, *p* < .001), 1998 to 2000 (*β* = .59, *p* < .001). In the high parental education group, there were positive and significant carry-over stability effects in self-reported health from 1990 to 1991 (*β* = .25, *p* < .05), 1991 to 1992 (*β* = .25, *p* < .01), 1992 to 1993 (*β* = .31, *p* < .001), and 2000 to 2007 (*β* = .28, *p* < .01). The high parental education group showed positive and significant carry-over stability effects in smoking 1990 to 1991 (*β* = .75, *p* < .01), 1991 to 1992 (*β* = .67, *p* < .001), 1992 to 1993 (*β* = .76, *p* < .001), 1993 to 1995 (*β* = .51, *p* < .001), 1995 to 1996 (*β* = .75, *p* < .001), and 1998 to 2000 (*β* = .51, *p* < .05).

**Parental income**

The smoking and self-reported health intercepts were negatively and moderately correlated in the low (*r* = -.39, *p* < .01) and high (*r* = -.42, *p* < .05) parental education groups. Regarding within-person associations between smoking and self-reported health, the high parental education group showed significant, negative, and small correlations in 1992 (*r* = -.21, *p* < .05) and 1995 (*r* = -.27, *p* < .05). There were no significant within-person correlations between smoking and self-reported health in the low parental income group.

In the low parental income group, there were significant and positive carry-over stability effects in self-reported health from 1990 to 1991 (*β* = .42, *p* < .001), 1991 to 1992 (*β* = .24, *p* < .05), 1992 to 1993 (*β* = .27, *p* < .01), 1998 to 2000 (*β* = .26, *p* < .05), and 2000 to 2007 (*β* = .31, *p* < .01). The high parental income group showed significant and positive carry-over stability effects in self-reported health from 1991 to 1992 (*β* = .24, *p* < .05), 1995 to 1996 (*β* = .33, *p* < .01), and 2000 to 2007 (*β* = .41, *p* < .001). Carry-over stability effects in smoking were apparent from 1990 to 1991 (*β* = .61, *p* < .01), 1991 to 1992 (*β* = .50, *p* < .001), 1992 to 1993 (*β* = .67, *p* < .001), 1993 to 1995 (*β* = .61, *p* < .001), 1995 to 1996 (*β* = .82, *p* < .001), 1996 to 1998 (*β* = .75, *p* < .001),  and 1998 to 2000 (*β* = .77, *p* < .001) in the low parental income group. In the high parental income group, there were positive carry-over stability effects from 1990 to 1991 (*β* = .83, *p* < .001), 1991 to 1992 (*β* = .75, *p* < .01), 1992 to 1993 (*β* = .73, *p* < .001), 1993 to 1995 (*β* = .56, *p* < .01), and 1995 to 1996 (*β* = .78, *p* < .001).
